# Evidence of positively selected G6PD A‐ allele reduces risk of *Plasmodium falciparum* infection in African population on Bioko Island

**DOI:** 10.1002/mgg3.1061

**Published:** 2019-12-24

**Authors:** Xue‐Yan Liang, Jiang‐Tao Chen, Yan‐Bo Ma, Hui‐Ying Huang, Dong‐De Xie, Santiago‐m Monte‐Nguba, Carlos Salas Ehapo, Urbano Monsuy Eyi, Yu‐Zhong Zheng, Xiang‐Zhi Liu, Guang‐Cai Zha, Li‐Yun Lin, Wei‐Zhong Chen, Xia Zhou, Min Lin

**Affiliations:** ^1^ School of Food Engineering and Biotechnology Hanshan Normal University Chaozhou Guangdong Province People’s Republic of China; ^2^ Department of Medical Genetics Shantou University Medical College Shantou Guangdong Province People’s Republic of China; ^3^ The Chinese Medical Aid Team to the Republic of Equatorial Guinea Guangzhou Guangdong Province People’s Republic of China; ^4^ Department of Medical Laboratory Huizhou Central Hospital Huizhou Guangdong Province People’s Republic of China; ^5^ School of Mathematics and Statistics Hanshan Normal University Chaozhou Guangdong Province People’s Republic of China; ^6^ Department of Medical Laboratory Malabo Regional Hospital Malabo Equatorial Guinea; ^7^ Department of Medical Laboratory Chaozhou People’s Hospital Affiliated to Shantou University Medical College Chaozhou Guangdong Province People’s Republic of China

**Keywords:** EHH, G6PD (A‐) deficiency, malaria, natural selection

## Abstract

**Background:**

Glucose‐6‐phosphate dehydrogenase (G6PD) is an essential enzyme that protects red blood cells from oxidative damage. Although G6PD‐deficient alleles appear to confer a protective effect of malaria, the link with clinical protection against *Plasmodium* infection is conflicting.

**Methods:**

A case–control study was conducted on Bioko Island, Equatorial Guinea and further genotyping analysis used to detect natural selection of the G6PD A‐ allele.

**Results:**

Our results showed G6PD A‐ allele could significantly reduce the risk of *Plasmodium falciparum* infection in male individuals (adjusted odds ratio [AOR], 0.43; 95% confidence interval [CI], 0.20–0.93; *p* < .05) and homozygous female individuals (AOR, 0.11; 95% CI, 0.01–0.84; *p* < .05). Additionally, the parasite densities were significantly different in the individuals with different G6PD A‐ alleles and individual levels of G6PD enzyme activity. The pattern of linkage disequilibrium and results of the long‐range haplotype test revealed a strong selective signature in the region encompassing the G6PD A‐ allele over the past 6,250 years. The network of inferred haplotypes suggested a single origin of the G6PD A‐ allele in Africans.

**Conclusion:**

Our findings demonstrate that glucose‐6‐phosphate dehydrogenase (G6PD) A‐ allele could reduce the risk of *P. falciparum* infection in the African population and indicate that malaria has a recent positive selection on G6PD A‐ allele.

## Graphical abstract

In this work, we firstly analysed a large case–control study of 342 malaria cases and 1,287 health controls on Bioko Island, Equatorial Guinea. Our findings demonstrate G6PD A‐ allele could reduce the risk of *Plasmodium falciparum* infection in African population and indicate malaria has recent positive selection on G6PD A‐ allele.

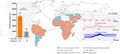

## 1 INTRODUCTION

Malaria is a major cause of mortality and morbidity worldwide. According to the 2018 World Malaria Report, there were approximately 219 million (95% confidence interval [CI]: 203–262 million) cases of malaria and an estimated 435,000 deaths in 2017, mostly among African children (Tajima, 1989). As a major cause of human morbidity, malaria has long been known to be the strongest selective pressure on the human genome over the past 10,000 years. This is the proposition of the “malaria hypothesis”, which posits that certain human genetic polymorphisms, especially those affecting red blood cells (RBCs), have been selected at high frequencies because they provide protection against the effects of malarial infections (Carter & Mendis, 2002).

Glucose‐6‐phosphate dehydrogenase (G6PD) is a key enzyme in the pentose phosphate pathway that plays an important role in the body's oxidative defences by governing the formation of nicotinamide adenine dinucleotide phosphate‐oxidase (NADPH) from nicotinamide adenine dinucleotide phosphate (Beutler & Duparc, 2007). It could protect RBCs from the harmful effects of reactive oxygen species. In the enzyme's deficient state (G6PD deficiency), only limited amounts of NADPH may generate, rendering the RBCs sensitive to oxidative damage, which may result in severe haemolytic episodes and in newborns with extreme hyperbilirubinemia and bilirubin encephalopathy. Currently, approximately 500 million people carry a deficient allele in the G6PD gene worldwide (Beutler & Duparc, 2007).

The view that G6PD deficiency has risen to high frequency in malaria‐endemic regions because of the resistance to malaria (Allison, 1960; Luzzatto, 2015) was proposed over half a century ago and is now widely accepted. However, the results of efforts to confirm that G6PD deficiency is protective in case–control studies and in vitro parasite culture experiments have been conflicting (Friedman, 1979; Guindo, Fairhurst, Doumbo, Wellems, & Diallo, 2007; Roth, Raventos‐Suarez, Rinaldi, & Nagel, 1983; Usanga & Luzzatto, 1985) This is partly due to the genetic complexity of the locus, which is X‐linked with multiple deficiency alleles, resulting in extensive allelic and phenotypic heterogeneity (Clarke et al., 2017). Additionally, if we looked at the association of G6PD deficiency and malaria susceptibility, the studies were more inconsistent, showing that G6PD deficiency was protective in heterozygous females (Bienzle, Ayeni, Lucas, & Luzzatto, 1972; Bienzle, Guggenmoos‐Holzmann, & Luzzatto, 1979; Clark et al., 2008; Ruwende et al., 1995) or had no effect on the susceptibility to uncomplicated malaria in either hemizygous males or heterozygous females (Enevold et al., 2005). Conversely, other studies showed an increase in the incidence of uncomplicated malaria in females heterozygous for G6PD deficiency (Lell et al., 1999; Parikh, Dorsey, & Rosenthal, 2004).

Bioko Island, Equatorial Guinea, is one of the areas with the highest level of malaria transmission in the world (Cook et al., 2018). Our previous epidemiological screening of inherited blood disorders revealed the common G6PD deficiency genotype in the Bioko population was G6PD A‐ (V68M and N126D), with 8.7% prevalence (Lin et al., 2015). Thus, we carried out this investigation to search for the evidence of natural selection of the G6PD A‐ allele by *Plasmodium falciparum* in the African population on Bioko Island.

## 2 MATERIALS AND METHODS

### 2.1 Sample collection

From April 2015 to October 2018, cases (*n* = 342) were selected from residents of Malabo who were diagnosed with *P. falciparum* malaria in earlier studies. Cases were defined as individuals who had at least one episode of *P. falciparum* malaria in the previous 2 years. Controls (*n* = 1,287) had no known episodes of malaria during the same 2 years of observation. All of cases and controls were selected from unrelated subjects. Health information was obtained from the record system of Malabo Regional Hospital and the outpatient clinic of the Chinese medical aid team.

Ethical approval was obtained from the Ethics Committee of Malabo Regional Hospital, the Ethics Committee of Shantou University Medical College. All subjects or their parents gave their informed consent. Blood samples were collected with EDTA‐K_2_ anti‐coagulated tubes from each subject, and 200 μL of blood was adsorbed on filter paper (Whatman 3 mm; GE Healthcare). These blood spots were air dried and stored individually in Ziploc bags containing silica desiccant beads and refrigerated (−20°C).

### 2.2 Parasite assessment

Thick and thin films were made on the same slide for each patient. The stained films were then examined microscopically for malaria parasites. Malaria parasite densities were calculated by counting the number of asexual parasites against 200 leukocytes in the same high power fields, assuming a leukocyte count of 8,000 white cells/µL. For quality control, archived malaria‐positive microslides were reexamined, and parasitemia was recorded. The *Plasmodium* species were identified using real‐time PCR followed by high‐resolution melting (HRM) (Wang et al., 2011). The pGEM‐T standard plasmids of four human *Plasmodium* species, including *P. falciparum*, *P. ovale*, *P. malariae* and *P. vivax*, which were provided by Dr Cao J (Jiangsu Institute of Parasitic Diseases) (Wang et al., 2011), were used as controls.

### 2.3 DNA extraction

Genomic DNA was extracted from all dried blood spots (*n* = 1,629) by the TIANamp blood spots DNA Kit (TIANGEN). The DNA concentration was measured by a Thermo Scientific Nanodrop‐2000 spectrophotometer and then adjusted to 50 ng/μL. The extraction protocol for filter paper samples was performed, and all samples were processed in the same way. Extracted DNA was stored at −20°C until testing by matrix‐assisted laser desorption/ionization time of flight mass spectrometry (MALDI‐TOF‐MS) and PCR‐HRM.

### 2.4 Genetic analysis for G6PD genotype

G6PD A‐: (V68M/N126D; c.202G>A/c.376A>G) was detected in the Bioko population (*n* = 1,629) by PCR‐HRM (Lin et al., 2015). The genomic sequence of the G6PD gene (GRCh38.p12: GCF_000001405.38) from NCBI (://www.ncbi.nlm.nih.gov) was used in the analysis. We designed the primer and 3’‐block probe (LunaProbe) (a C3 spacer phosphoramidite attached to a standard 3′ CpG) for G6PD A‐ (Table S1). And more detailed information about PCR reaction and HRM analysis for G6PD genotyping can be found in Data S1.

### 2.5 Analysis of G6PD enzyme activity

In our study, due to resource constraints, only the female malaria cases carrying the G6PD A‐ allele (*n* = 20) were recalled to detect phenotypic G6PD deficiency by a commercial fluorescence spot test kit (Guangzhou Micky Medical Instrument Co.) after molecular diagnosis, as previous reported (Lin et al., 2015; Yang et al., 2015). The cut‐off value for this study was set at 2.7 U/gHb (Yang et al., 2015). The assay was performed according to the manufacturer's protocol. Then, an aliquot of the lysate of these suspected deficient samples (G6PD value <2.7 U/gHb) was spotted on filter paper (Whatman 3 mm; GE Healthcare), air dried and examined under UV light. Samples from G6PD‐deficient subjects showed no or diminished fluorescence compared with nondeficient samples.

### 2.6 Evolutionary analysis

#### 2.6.1 SNP selection

A 2.4‐Mb region encompassing the human G6PD gene was screened for appropriate haplotype‐tag SNPs (Tag SNPs). Data from the African population from 1000 Genomes (http://grch37.ensembl.org/index.html) were used. Tag SNPs were selected based on their minor allele frequency (MAF ≥0.05), and the Tag SNPs were assessed using Haploview version 4.2 software under the parameter *r* ^2^ ≥ 0.8 (Barrett, Fry, Maller, & Daly, 2005). To reduce the genotyping cost, just one or two SNPs were selected from each block or boundary. As a result, a total of 31 Tag SNPs were determined (Table S2).

#### 2.6.2 Tag SNP genotyping

A panel of 192 individuals, randomly selected from the general population of 1,629 individuals, were genotyped for the 31 Tag SNPs. Genotyping of Tag SNPs was performed by using the Sequenom MassARRAY IPLEX platform, which could detect multiple SNPs at the same time (Buetow et al., 2001). Primer design, synthesis, and genotype testing were conducted by Bioyong Technologies Inc., according to standard laboratory procedures. For quality control, 5% of DNA samples were randomly selected for duplicate tests, and the concordance rate was as high as 100%.

#### 2.6.3 Data analysis

Hardy–Weinberg equilibrium (HWE) of each Tag SNP was assessed in the population (*n* = 192), and the thre**s**hold *p* < .001 was regarded to indicate deviation from HWE. Haplotype was inferred by a Bayesian statistical method with Phase version 2.1 software using the default parameter set with 1,000 iterations. The linkage disequilibrium (LD) values between the Tag SNPs were calculated using Haploview version 4.2 software. The extended homozygosity haplotype (EHH) values were calculated with the R package rehh (Gautier, Klassmann, & Vitalis, 2017). We further analyzed the data from the 1000 Genomes Project (http://www.internationalgenome.org/) on the X‐chromosome SNPs (chrX:153564217‐153964217) from all African populations worldwide. The EHH around the core SNP (rs1050828) and integrated haplotype score (iHS) based on the standardized log ratio of the integrals of the observed decay of EHH with the minor allele frequency >5% were computed with the rehh package. An average recombination rate within the entire G6PD region was calculated with the R package Ldheatmap (Vens & Ziegler, 2017). To obtain the crude estimate of the ages of the sweeps, we performed the approach introduced by Voight, Kudaravalli, Wen, and Pritchard (2006). The inferred haplotype network was constructed by a median‐joining method with Network version 5.0.0.3 software using the default settings.

### 2.7 Statistical methods

All statistical analyses were performed using SPSS version 19.0 software (SPSS Inc.). We conducted univariate and multivariate analyses using unconditional logistic regression and adjusting for prespecified potential confounding factors between cases and controls to evaluate the association between G6PD A‐ genotypes and the risk *P. falciparum* infection; those factors included in the final unconditional multivariate logistic regression model for the primary outcome were age group and sex. All results are expressed as odds ratios (OR), adjusted odds ratios (AOR) with 95% CIs. *p* < .05 indicated statistical significance. To investigate the association of G6PD genotypes and parasite burden among subjects with malaria, we examined parasite density in different G6PD genotype groups by nonparametric Mann–Whitney *U* test. *p* < .05 indicated statistical significance.

## 3 RESULTS

### 3.1 Population information

The information of the subjects in our study, including 342 cases and 1,287 controls, is shown in Table 1. A total of 226 subjects were found to have genotypic G6PD A deficiency by PCR‐HRM. The melting curve profiles for G6PD A‐ (V68M and N126D) are shown in Figures S1 and S2, respectively. There were statistically significant differences in the frequency of age between cases and controls (*p* < .05). Therefore, we need to perform statistical correction of the age between them.

**Table 1:** Characteristics of cases and controls

Variable	Case (*n* = 342)	Control (*n* = 1,287)	*p* value
Mean age (year)	30.8	27.5	
Age group (year), *n*/*N* (%)			<.001
1–9	41 (11.9)	27 (2.1)	
10–24	101 (29.4)	396 (30.8)	
25+	200 (58.5)	864 (67.1)	
Gender, *n*/*N* (%)			.982
Male	161 (47.1)	605 (47.0)	
Female	181 (52.9)	682 (53.0)	
G6PD A‐ genotype, *n*/*N* (%)			.003
Wild type	314 (91.8)	1,089 (84.6)	
Hemizygote	8 (2.3)	69 (5.4)	
Heterozygote	19 (5.6)	103 (8.0)	
Homozygote	1 (0.3)	26 (2.0)	

Abbreviation: G6PD, glucose‐6‐phosphate dehydrogenase.

### 3.2 G6PD A‐ deficiency and malaria

There was a lower frequency of genotypic G6PD A‐ deficiency among cases (28/342, 8.19%) than controls (198/1,287, 15.38%) (Pearson *χ* ^2^ value = 9.2036, *p* = .002). This result indicated that genotypic G6PD A deficiency provided host protection for *P. falciparum* infection. The unconditional logistic regression was used to evaluate the possible effect factors for *P. falciparum* infection. The effects of genotypic G6PD A deficiency, sex and age group on the frequency of malaria are shown in Table S3. Consistent with the above result, it also demonstrated a significant protection in hemizygous male individuals and homozygous female individuals with AOR 0.43 (95% CI, 0.20–0.93; *p* < .05) and AOR 0.11 (95% CI, 0.01–0.84; *p* < .05), respectively.

**Figure 1:**
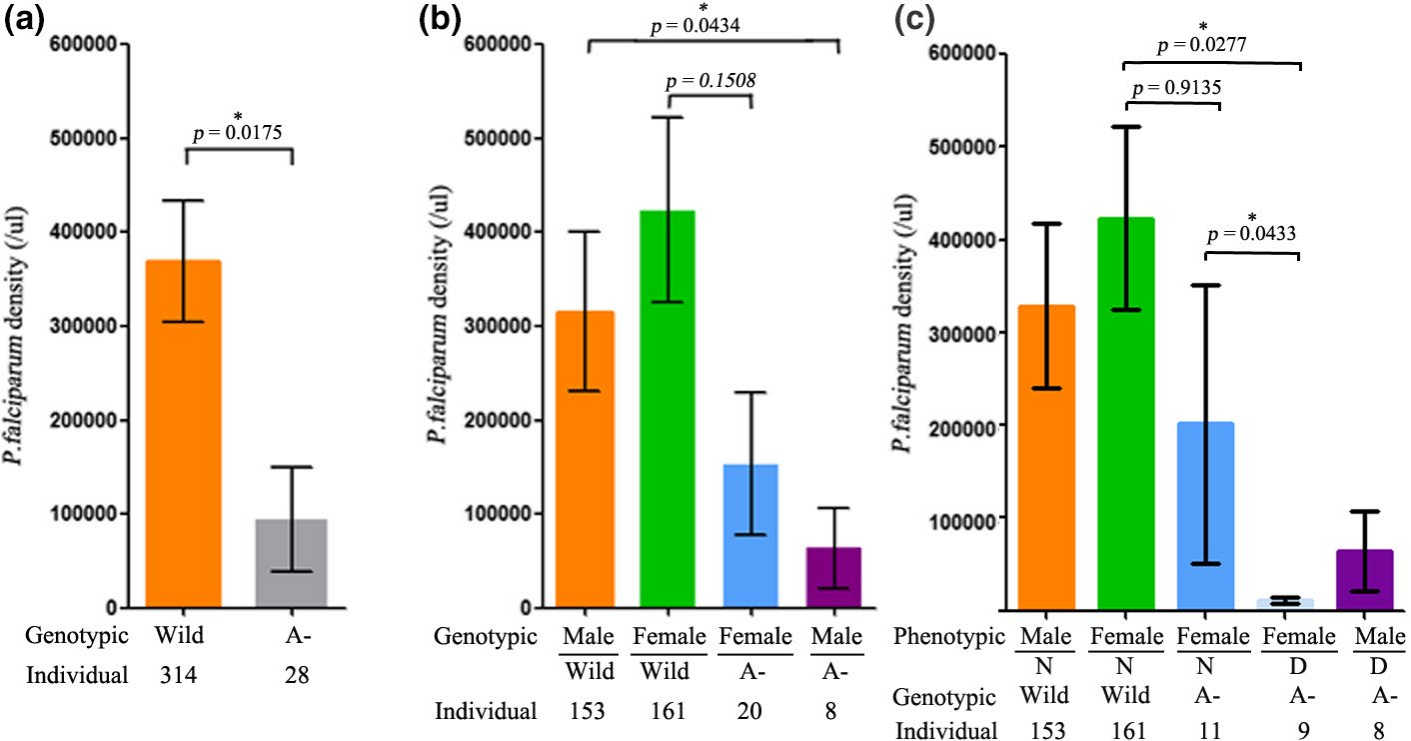
Effect of G6PD A‐ carrier status on parasite densities. (a) *Plasmodium falciparum* parasite densities (means ± *SD*) according to G6PD A‐ carrier status (orange, individuals with wild type [without G6PD A‐ allele]; gray, individuals with G6PD A‐ allele). (b) *Plasmodium falciparum* parasite densities (means ± *SD*) according to gender and G6PD A‐ carrier status (orange, male individuals with wild type; green, female with wild type; blue, female individuals with G6PD A‐ allele; purple, male with G6PD A‐ allele). (c) *Plasmodium falciparum* parasite densities (means ± *SD*) according to gender, G6PD A‐ carrier status and enzyme activity (orange, male individuals with wild type; green, female with wild type; blue, female individuals carried G6PD A‐ allele [≥2.7 U/gHb]; baby blue, female individuals carried G6PD A‐ allele [<2.7 U/gHb]; purple, male individuals carried G6PD A‐ allele [<2.7 U/gHb]). G6PD, glucose‐6‐phosphate dehydrogenase

A G6PD A‐ allele dose effect on *P. falciparum* density was observed. From Figure 1, we found that the parasite density tended to be lower in the individuals carrying the G6PD A‐ allele than in those without the G6PD A‐ allele (*p* = .0175). By separating the individuals into female and male groups, different analysis strategies were implemented. In the male group, the parasite density was significantly lower in hemizygous individuals (*n* = 8) than in those without the G6PD A‐ allele (*p* = .0434) (Figure 1b). Although the parasite density was lower in female individuals with the G6PD A‐ allele (*n* = 20; 19 heterozygote and 1 homozygote) than in those without, no significant difference was observed between them (*p* = .1508). To investigate the effect of the level of enzyme activity on parasite density in heterozygous female individuals, the female heterozygotes were classified based on the cut‐off value (<2.7 U/gHb). As shown in Figure 1c, the parasite density was significantly lower in female individuals with a value <2.7 U/gHb (*n* = 9; 8 heterozygote and 1 homozygote) than those with a value ≥2.7 U/gHb (*n* = 11) (*p* = .0433) or females without the G6PD A‐ allele (*p* = .0277).

### 3.3 Extended LD around G6PD A‐ allele

**Figure 2:**
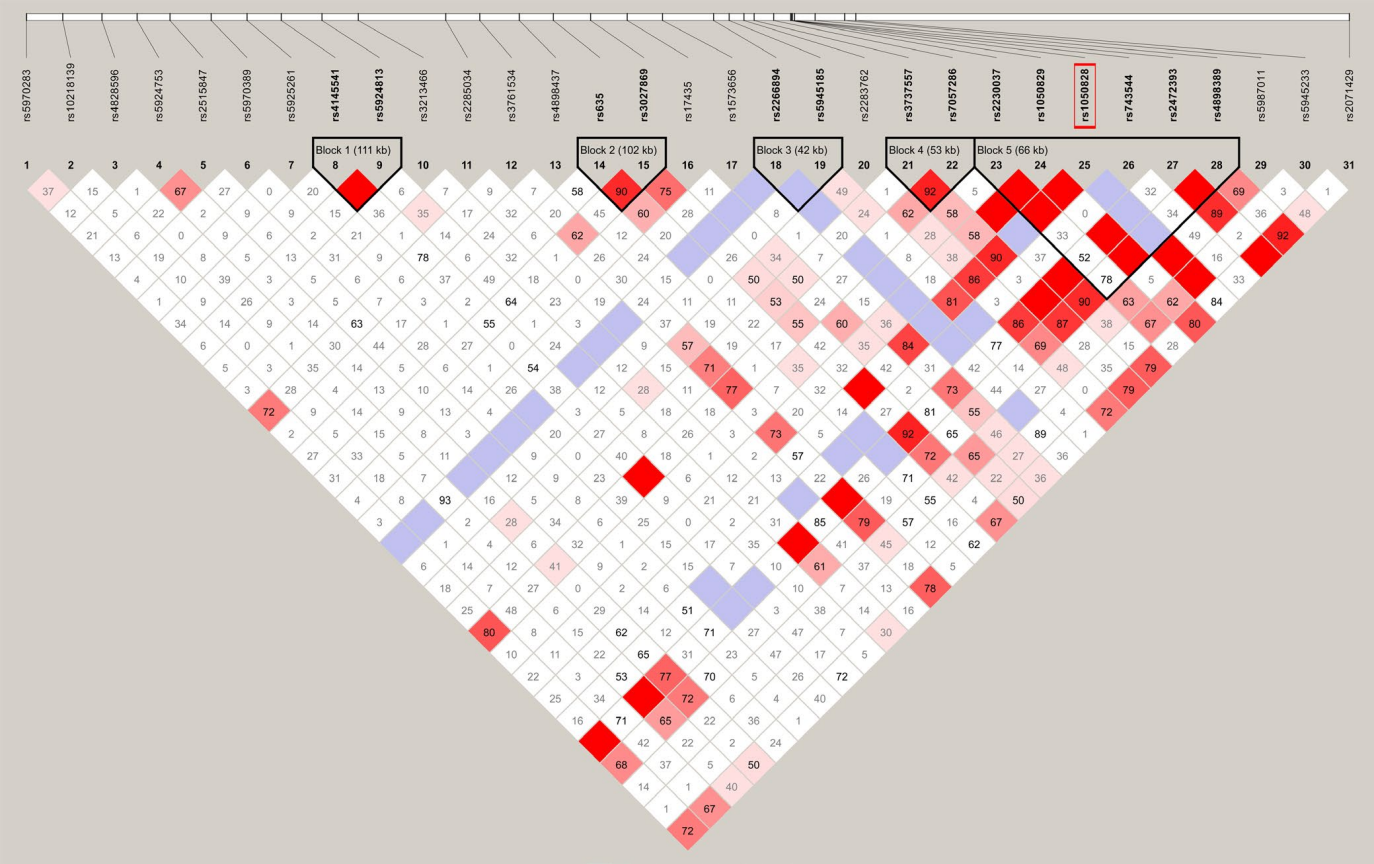
LD structure constructed from 31 marker‐inferred haplotypes in African on Bioko Island, Equatorial Guinea. The red rectangle indicates the position of the G6PD A‐ allele (rs1050828). The value in the square is the |*D*’| between the pair of loci. Darker red squares indicate higher values of |*D*’| with statistical significance (LOD >2). Blue squares indicate high values of |*D*’| but with no statistically significant LD. White squares indicate low values of |*D*’| and LOD simultaneously. The black triangle indicates the LD block based on the four gamete rule method. G6PD, glucose‐6‐phosphate dehydrogenase; LD, linkage disequilibrium

**Figure 3:**
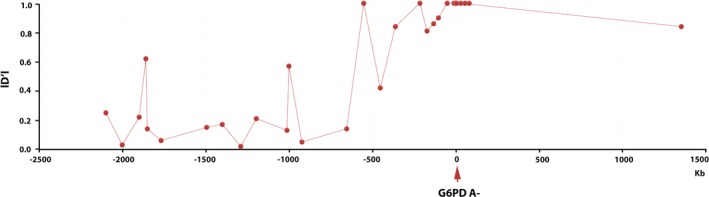
Pairwise |*D*’| between the G6PD A‐ allele (rs1050828) and the other 30 Tag SNPs. The *x*‐axis represents the relative position to the G6PD allele (arrow at 0 kb), and the black dots indicate the distance and |*D*’| of corresponding markers. EHH, G6PD, glucose‐6‐phosphate dehydrogenase

To examine the pattern of LD in the 2.4‐Mb region encompassing the human G6PD gene, all possible pairwise |*D*’| values (Lewontin, 1964) among the Tag SNPs were calculated in the random population (*n* = 192). The detailed data are listed in Table S2. Pairwise comparisons showed a strong LD within Tag SNPs, which was consistent with theoretical expectations (Figure 2). A 66Kb block was formed among the Tag SNPs around the G6PD A‐ allele based on the four gamete rule method, indicating a hitchhiking effect due to linkage with the G6PD A‐ allele (Figure 2). In addition, the G6PD A‐ allele showed a high degree of LD with upstream and downstream Tag SNPs at a distance of approximately 200 kb, including rs1050829, rs2230037, rs7057286, rs3737557, rs2283762, rs5945185, rs1573656, rs743544, rs2472393, rs5987101, and rs5945233 (although some of them had no statistical significance) (Figure 3).

### 3.4 Test for recent selection on G6PD A‐ allele

**Figure 4:**
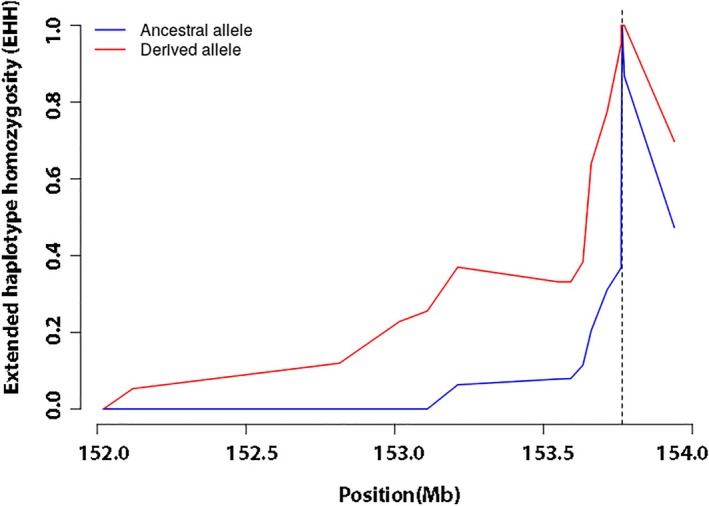
EHH, the decay of extended haplotype homozygosity in the African on Bioko Island, Equatorial Guinea. EHH was computed for the ancestral and derived alleles at the focal SNP (rs1050828) of 31 Tag SNPs over the ~200 kb region of the G6PD gene. EHH, extended homozygosity haplotype; G6PD, glucose‐6‐phosphate dehydrogenase

**Figure 5:**
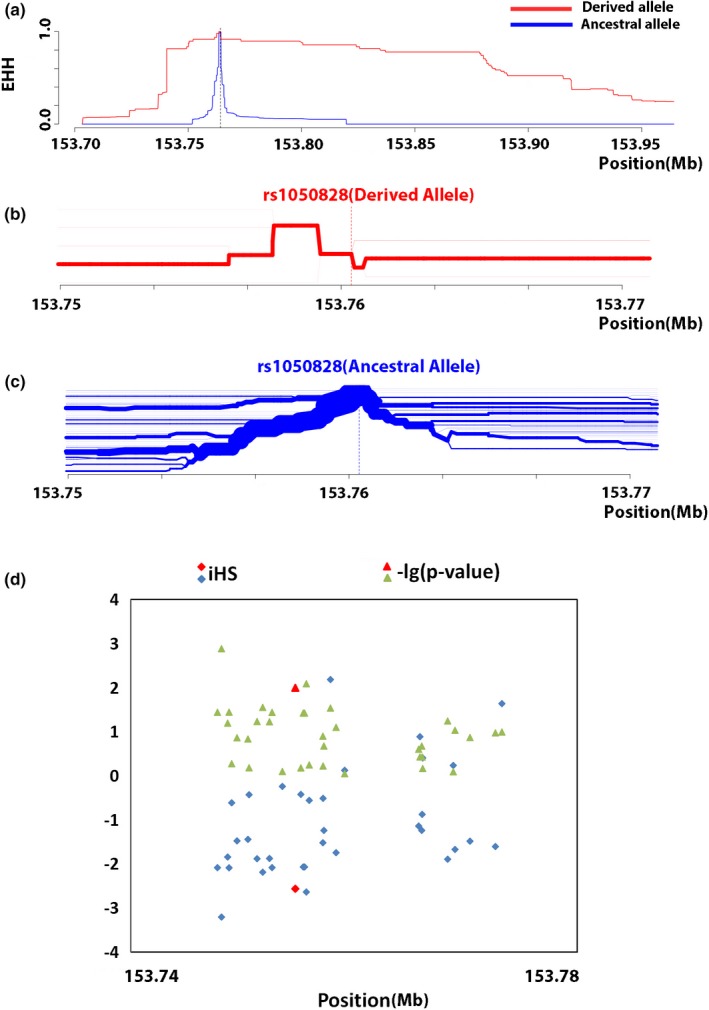
Additional data analysis of G6PD A‐ allele in African populations from the 1000 Genomes Project in the G6PD region. (a) EHH at varying distances from the core SNP (rs1050828). (b, c) Haplotype bifurcation diagrams drawn for the ancestral and derived allele of the core SNP. (d) Plots of iHS values and *p*‐values for all SNPs with minor allele frequencies >5%. The red color indicates the core SNP (rs1050828). EHH, extended homozygosity haplotype; G6PD, glucose‐6‐phosphate dehydrogenase

To detect the recent selection on the G6PD A‐ allele, the EHH was computed around the core SNP, EHH equals 1 at the focal SNP and decays monotonically to 0 as one moves away from the focal SNP (Voight et al., 2006). As shown in Figure 4, the EHH of the 31 Tag SNPs decays far more rapidly for the haplotypes carrying the ancestral variant at the core SNP than for those carrying the derived variant. Since our data consisted of preascertained SNPs, which lack some data on the right side of the core SNP (Figure 4), a further bioinformatic analysis containing more information from the 1000 Genomes Project was developed to verify the recent selection on the G6PD A‐ allele. We observed the same result in that the haplotype carrying the ancestral allele decayed far more rapidly than those carrying the derived allele (Figure 5a). The bifurcation diagram in Figure 5b,c provides another visualization of the structuring of haplotype diversity around the focal SNP at position 153,764,217 bp, representing the breakdown of LD at increasing distance from the focal SNP, which shows a high level of extended homozygosity in the G6PD A‐derived allele variant. We plotted the value of iHS and *p*‐value for all SNPs with a minor allele frequency >5% covering the G6PD region, and a high negative value of iHS was found around the core SNP (rs1050828), which shows evidence for selection (Figure 5d).

### 3.5 Age estimates of the G6PD A‐ allele

We estimated the crude age of the G6PD A‐ allele using the method described by Voight et al. with the data of EHH and recombination distances (*r*). The local rate of recombination in this region of the X‐chromosome is ~1.50 cM/Mbp, which contributes to the estimation of recombination distances. According to Voight et al., *Pr[Homoz] *= e*^−2rg^*, where *Pr[Homoz]* is the probability that two chromosomes are homozygous at a recombination distance *r* from the selected site, given a common ancestor *g* generations before the present. Taking the generation time to be 25 years, the ancestor time in years becomes *t* = 25 *g*. For the G6PD A‐ allele in the African population, we observed *g* = 250, which means an estimation age of the G6PD A‐ allele is 6,250 years (if *t* = 30 *g*, the age is 7,500).

### 3.6 Origin of the G6PD A‐ allele in the Bioko population

**Figure 6:**
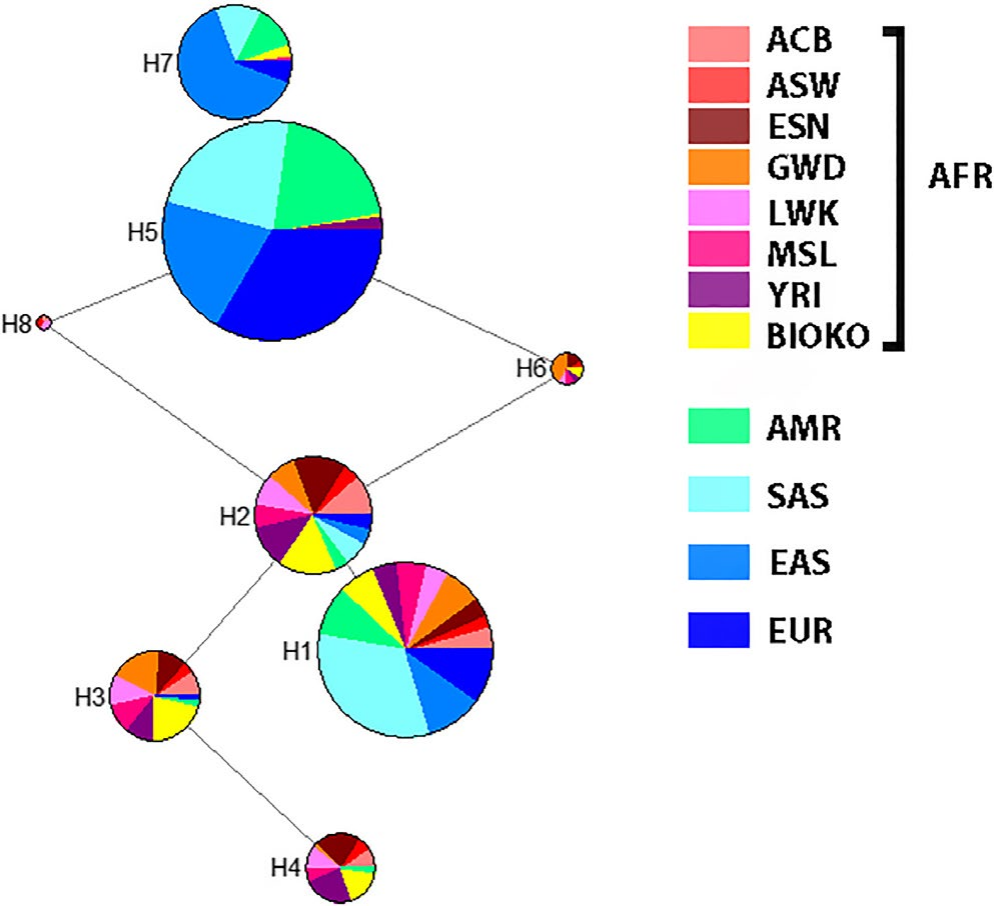
Phylogenetic network of haplotypes of five SNPs and the G6PD A‐ allele. The five SNPs used to construct the network were rs2230037, rs1050829, rs743544, rs2472393, and rs4898389. H1, ATCGTG; H2, GTCGTG; H3, GCCGTG; H4, GCTGTG; H5, GTCGTA; H6, GTCGTA; H7, GTCACA; H8, GTCGCG. For each haplotype, the underlined SNP represents the G6PD A‐ allele. ACB, African Caribbean in Barbados; AMR, American; ASW, Americans of African Ancestry in SW USA; EAS, East Asian; ESN, Esan in Nigeria; EUR, European; G6PD, glucose‐6‐phosphate dehydrogenase; GWD, Gambian in Western Divisions in Gambia; LWK, Luhya in Webuye, Kenya; MSL, Mende in Sierra Leone; SAS, South Asian; YRI, Yoruba in Ibadan, Nigeria

To reconstruct the evolutionary history of the A‐ in the Bioko population and other African populations, we collected polymorphic site information including the G6PD A‐ allele and five adjacent SNPs, which formed an ~66 kb LD block (Figure 2), from different male populations in the 1000 Genomes Project (://grch37.ensembl.org) for haplotype analysis. A network of haplotypes was constructed (Figure 6). Of the eight haplotypes worldwide, seven haplotypes were found in the Bioko population. Only one haplotype carried the G6PD A‐ allele (H4: GCTGTG), and it arose from the H3 (GCCGTG) haplotype by a single mutation (Figure 6). This haplotype (H3) was common in the Bioko population (23.4%) and prominent in AFR populations (ACB, ASW, ESN, GWD, LWK, MSL, and YRI), but barely seen in the rest of the world, with a few exceptions in AMR (1.2%; only 2 in 169 chromosomes) (Figure 6).

## 4 DISCUSSION

As a major cause of human morbidity, malaria parasites have had a profound selection pressure on recent human evolution (Carter & Mendis, 2002), and the geographical distribution of several RBC genetic polymorphisms that are protective against malaria is consistent with this theory. In contrast to other RBC genetic polymorphisms, the observed associations between malaria and G6PD deficiency were inconsistent at lower levels of statistical significance and varied widely among the study sites. This was partly because of the genetic complexity of G6PD deficiency, which was affected by multiple allelic variants and has different effects in males and females. Therefore, we adopted a simplified strategy to solve the problem as follows: (a) Because of the effect of geographic isolation, genetic variation was lower on average on islands than in mainland populations (Lin et al., 2015). Therefore, an island population was selected to reduce the genetic complicating factors of the case–control study. Our previous study revealed that the type of G6PD‐deficient allele of the Bioko population was relativity simplification (Lin et al., 2015); (b) As a result of X‐chromosome inactivation, phenotypic and genotypic results were consistent in hemizygous male individuals and homozygous female individuals and were usually inconsistent in the heterozygous female individuals. Heterozygous female individuals may be either phenotypically deficient or normal depending on the relative proportion of circulating deficient and nondeficient red cells because female individuals exhibit mosaicism in the expression of G6PD. The failure of the laboratory determination of G6PD phenotypic deficiency was another potential source of error. The proportion of inconsistent phenotypic and genotypic results could produce significant bias in the statistical results. Therefore, in our study, all individuals were identified by molecular methods instead of enzymatic analysis, thus enabling confirmation of every heterozygote in the population. Then, to understand the effect of the degree of G6PD deficiency in heterozygous females, the heterozygous female individuals were stratified according to their level of enzyme activity in the case group.

Based on the strategy, our results provided positive evidence that the G6PD A‐ allele could reduce the prevalence of *P. falciparum* infection (Table S3, *p* < .001) and inhibit parasite density in vivo (Figure 1a, *p* = .0175). This was consistent with the results of a meta‐analysis by Mbanefo et al. (2017). They observed a negative association between G6PD and uncomplicated *P. falciparum* malaria from a total of 12 studies performed in Africa (OR, 0.59; 95% CI, 0.40–0.86; *n*, 11; *p* = .007) (Voight et al., 2006). It was also supported by the recently published finding that G6PD Mediterranean deficiency conferred significant protection against *P. vivax* malaria infection in Pakistan (Leslie et al., 2010). In contrast to those studies that showed that only female heterozygous individuals were protected against malaria, (Luzzatto, 2015; Uyoga et al., 2015) our study revealed that both male hemizygous individuals and phenotypically deficient female individuals (heterozygotes with G6PD value <2.7 U/gHb and homozygotes) could provide the host protection against malaria through decreasing the parasite density (Figure 1c). We thought that the level of G6PD enzyme activity may play a key role in this phenomenon. Luzzatto, Usanga, & Reddy (1969) studied the differential parasitisation of deficient and nondeficient RBCs of the same individual in 20 heterozygous females. They observed that parasitization was 2–80 times greater in nondeficient than in deficient cells (Luzzatto et al., 1969). This indicated that G6PD‐deficient cells were protective against malaria. Thus, homozygous females, hemizygous males, and phenotypically deficient heterozygous females should also be protected.

Evolutionary genetic analysis was used to identify the signatures of selection for the G6PD‐deficient A‐ allele in the human genome. According to the genotyping of 31 Tag SNPs around the G6PD A‐ allele and an additional analysis based on the 1000 Genomes Project, our results were consistent with the malaria hypothesis. There was a strong extended LD between the Tag SNPs located within a ~200 kb region of the G6PD gene. Additionally, the long‐range haplotype test showed a significantly high EHH value, indicating recent positive selection of the G6PD A‐ allele. Previous estimates for the age of the G6PD A‐ allele suggest that the allele is young (<20,000 years) on the basis of closely linked microsatellite variability, (Tishkoff et al., 2001) coalescent‐based analysis of a G6PD gene tree, (Coop & Griffiths, 2004) and intergenic LD (Saunders, Hammer, & Nachman, 2002; Saunders, Slatkin, Garner, Hammer, & Nachman, 2005). Our current analysis estimated that the G6PD A‐ allele arose within the past 6,250 (assuming a 25‐year generation time) or 7,500 years (assuming a 30‐year generation time) and originated from a single African ancestor. This age estimate was similar to the report of Tishkoff (Tishkoff et al., 2001) based on intra‐allelic microsatellite variability (3,840–11,760 years) and older than the one reported by Saunders et al. (2005) (2,500–3,750 years, assuming a 25‐year generation time). Additionally, this estimate was consistent with archaeological and historical documents indicating that malaria has had a significant impact on humans only within the past 10,000 years (Saunders et al., 2002; Tishkoff et al., 2001). Or, the parasite population went through a significant expansion approximately 6,000 years ago from one or a few genomes (Hume, Lyons, & Day, 2003; Joy et al., 2003). Likewise, the malaria‐protective human alleles that cause sickle cell disease and a‐thalassaemia also appear to have origins within this timeframe (Hume et al., 2003). The first evidence of a farming economy in Africa was 6,000–7,000 years ago in Egypt and is believed to have derived from Asia Minor (Hume et al., 2003; Tishkoff et al., 2001). The human slash‐and‐burn agricultural activity in Africa resulted in an increase in the population density of *Anopheles gambiae*, the mosquito vectors for *P. falciparum* (Wiesenfeld, 1967). Additionally, agriculture enabled increased human population density, facilitating the spread of malaria (Tishkoff et al., 2001).

In conclusion, our data provide strong empirical evidence that the G6PD A‐ allele could reduce the risk of *P. falciparum* infection in the African population and complement the evolutionary genetic finding of the balancing selection hypothesis of G6PD deficiency in previous studies.

## CONFLICT OF INTEREST

The Authors report no conflicts of interest.

## AUTHORS CONTRIBUTION

ML, X‐YL, and J‐TC designed the study. J‐TC, S‐mM‐N, CSE, UME, and D‐DX, collected the samples, entered the data and validated microscopy. X‐YL, Y‐BM, and H‐YH analyzed and interpreted the data. Y‐ZZ, X‐ZL, G‐CZ, L‐YL, W‐ZC, and XZ conducted the laboratory work (G6PD genotyping and analysis of G6PD enzyme activity). ML, J‐TC, and X‐YL wrote the paper. All authors critically reviewed the paper and approved the final version of the paper for submission.

## Data Availability

The data used to support the findings of this study are available from the corresponding author upon request.
