# Supplementary figures and images for: Evidence of positively selected G6PD A‐ allele reduces risk of Plasmodium falciparum infection in African population on Bioko Island
Source: Mol Genet Genomic Med. 2019 Dec 24;8(2):e1061. doi: 10.1002/mgg3.1061 (PMC7005621; doi:10.1002/mgg3.1061)

Melting Peaks

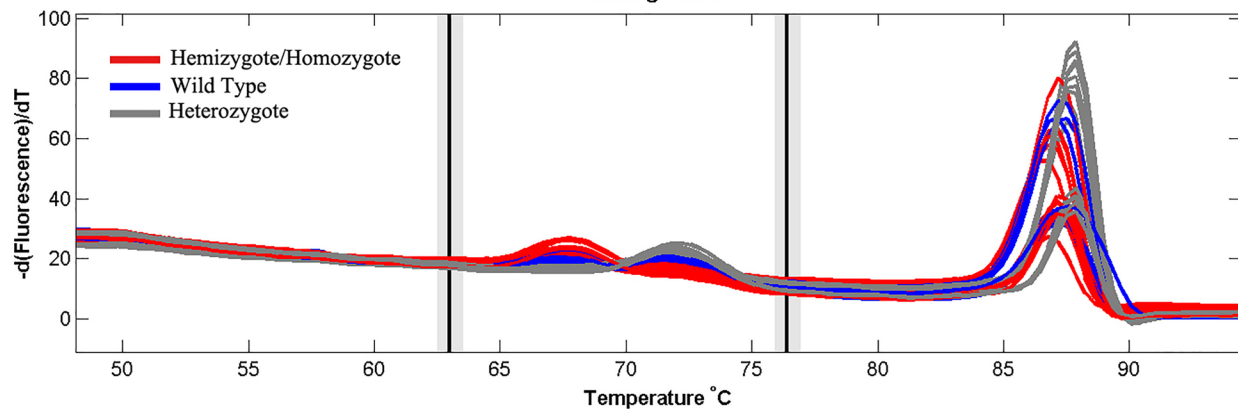

Normalized Melting Curves

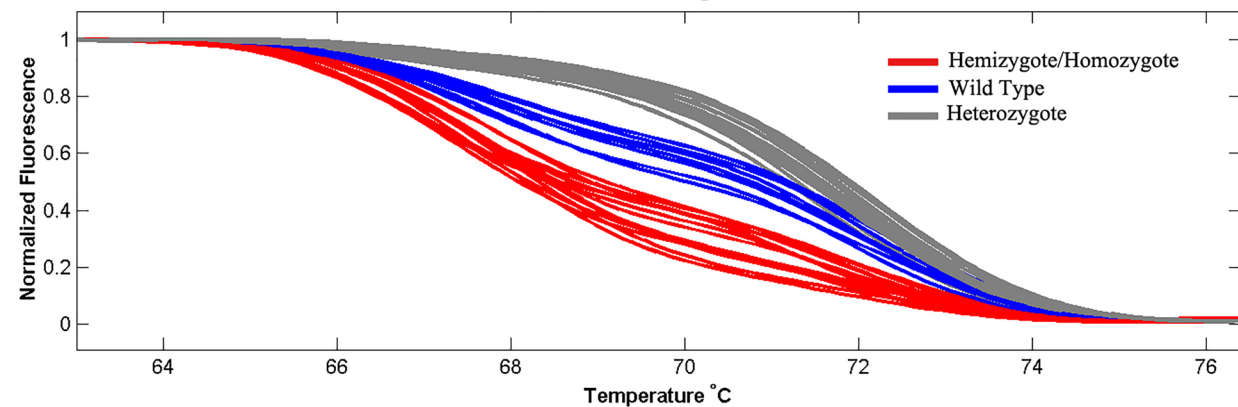

Normalized Melting Peaks

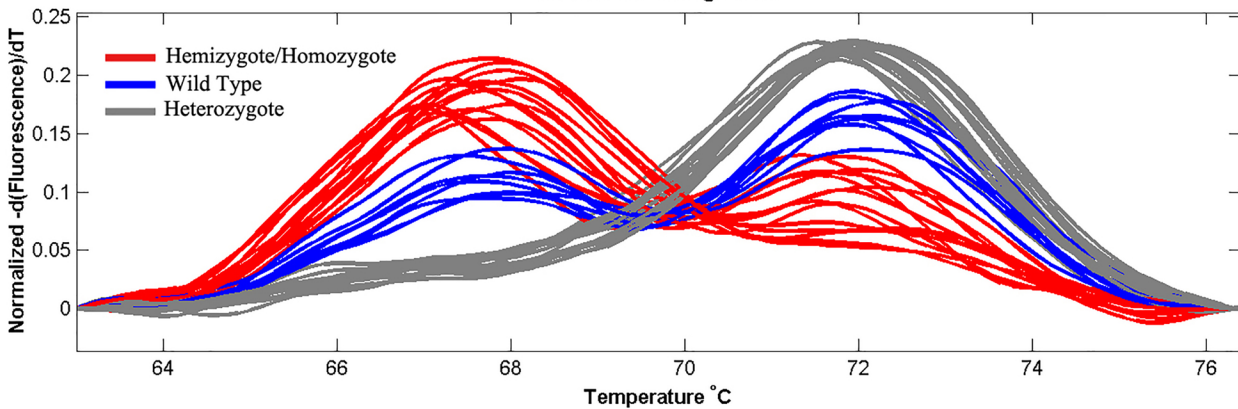

Supplement: Supplementary file 1 [file MGG3-8-e1061-s001.pdf]

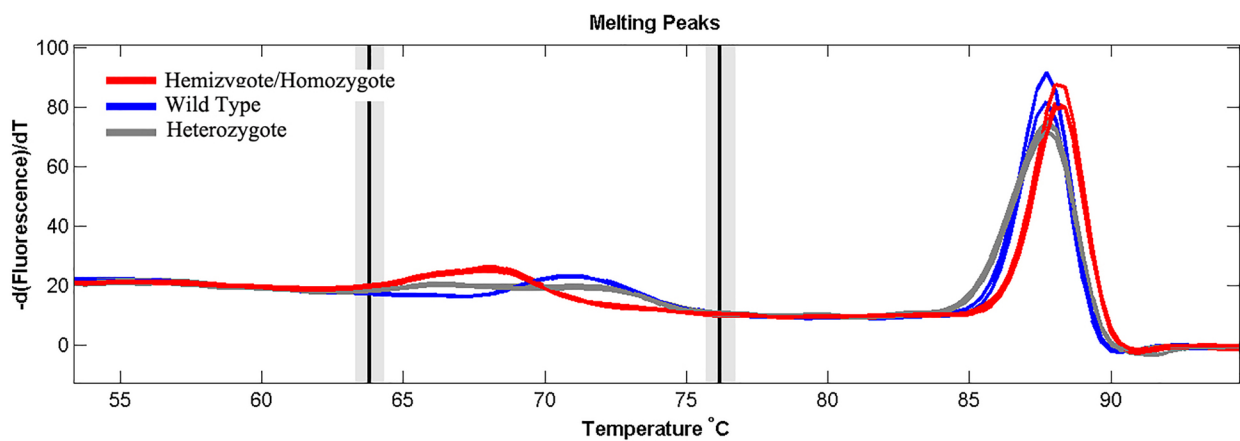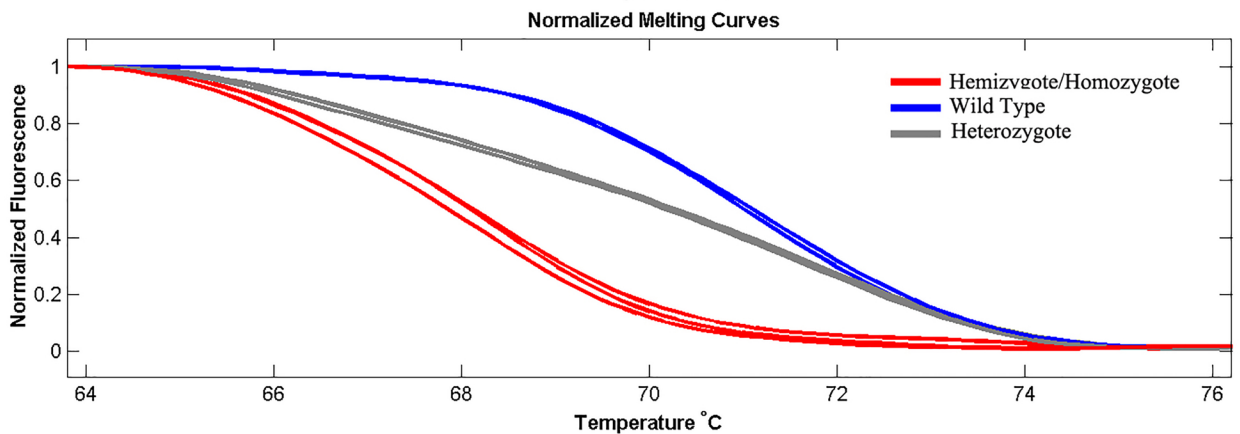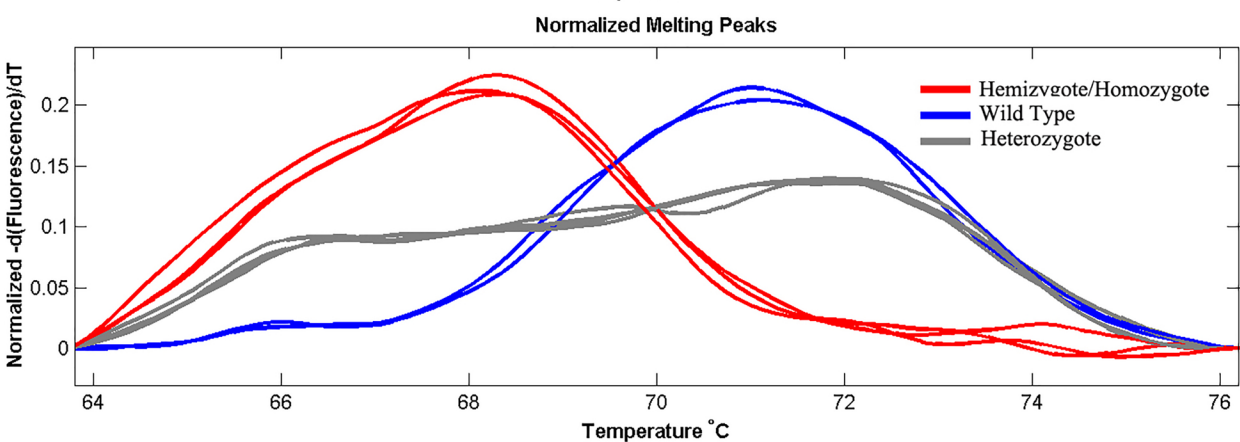

Supplement: Supplementary file 2 [file MGG3-8-e1061-s002.pdf]
