# Supplementary material for: Evidence of positively selected G6PD A‐ allele reduces risk of Plasmodium falciparum infection in African population on Bioko Island
Source: Mol Genet Genomic Med. 2019 Dec 24;8(2):e1061. doi: 10.1002/mgg3.1061 (PMC7005621; doi:10.1002/mgg3.1061)
Supplement: Supplementary file 3 [file MGG3-8-e1061-s003.docx]

**Supplemental Table 1. Primers and LunaProbes used for genotyping G6PD A-.**

|  | **NM_001042351.2:c.202G>A** | **NM_001042351.2:c.376A>G** |
| --- | --- | --- |
| Forward primer (5’-3’) | TGCCCTCAGGTGGCTGTT | ATGATGCAGCCTCCTACCAG |
| Reverse primer (5’-3’) | GCTCACTCTGTTTGCGGATGT | CCAGGTAGAAGAGGCGGTT |
| Probe (5’-3’) | GCCCGAAAACACCTTCATCGTGGGCTAT-block | ACAGCCACATGAATGCCCTCCACCTG-block |
