# Supplementary material for: Evidence of positively selected G6PD A‐ allele reduces risk of Plasmodium falciparum infection in African population on Bioko Island
Source: Mol Genet Genomic Med. 2019 Dec 24;8(2):e1061. doi: 10.1002/mgg3.1061 (PMC7005621; doi:10.1002/mgg3.1061)
Supplement: Supplementary file 5 [file MGG3-8-e1061-s005.docx]

**Supplemental Table 3** . Effect of genotypic G6PD A- deficiency, sex and age group on frequency of malaria.

| **Variable** | **Malaria history, *n*/N (%)** | **Odds Ratio (95% CI)** | ***p* value** | **Adjusted Odds Ratio (95% CI)** | ***p* Value** |
| --- | --- | --- | --- | --- | --- |
| **G6PD A- genotype** |  |  |  |  |  |
| Wild type | 314/1403 (22.38) | 1 |  | 1 |  |
| Hemizygote | 8/77 (10.39) | 0.40 (0.19-0.85) | 0.016 | 0.43 (0.20-0.93) | 0.031 |
| Heterozygote | 19/122 (15.57) | 0.64 (0.39-1.06) | 0.083 | 0.66 (0.39-1.12) | 0.128 |
| Homozygote | 1/27 (3.70) | 0.13 (0.02-0.99) | 0.049 | 0.11 (0.01-0.84) | 0.034 |
| **Gender** |  |  |  |  |  |
| Female | 181/863 (20.97) |  |  | 1 |  |
| Male | 161/766 (21.02) | 1.00 (0.79-1.27) | 0.982 | 1.00 (0.77-1.27) | 0.967 |
| **Age group (y)** |  |  |  |  |  |
| 25+ | 200/1064 (18.80) | 1 |  | 1 |  |
| 10-24 | 101/496 (20.36) | 1.09 (0.84-1.43) | 0.524 | 1.09 (0.83-1.43) | 0.533 |
| 1-9 | 41/68 (60.29) | 6.56 (3.94-10.91) | 0.000 | 6.63 (3.95-11.13) | 0.000 |
