# Supplementary material for: Evidence of positively selected G6PD A‐ allele reduces risk of Plasmodium falciparum infection in African population on Bioko Island
Source: Mol Genet Genomic Med. 2019 Dec 24;8(2):e1061. doi: 10.1002/mgg3.1061 (PMC7005621; doi:10.1002/mgg3.1061)
Supplement: Supplementary file 6 [file MGG3-8-e1061-s006.docx]

**Supplemental File 1**

**Genetic analysis for G6PD genotype**

PCR reaction conditions were optimized and performed on a 96-well microtiter plate system using the LifeECO gradient thermocycler (Bioer Technology Co. LTD, China), followed by HRM analysis on a LightScanner-96 (Idaho Technology, Inc., Salt Lake City, UT). PCR was carried out in a total volume of 20 µL, containing 2 µL of sample DNA, 12.5 µL of 2×Taq plus PCR MasterMix (Aidlab Co. LTD, China), with forward primers at a final concentration of 0.05 µM, reverse primers at a final concentration of 0.2 µM (asymmetric PCR), and allele specific probes at a final concentration of 0.2 µM and 1 µL LC Green plus (Idaho Technology). Standard controls (the identified DNA by Sanger sequencing) were used as reference genotypes for the HRM analysis. All DNA samples and standard controls were amplified simultaneously in two parallel reactions with each primer set and LunaProbes. The cycling conditions were the same for all amplicons as follows: 95°C for 3 min, followed by 40 cycles of 95°C for 30 s, 55°C for 10 s, 72°C for 20 s. Then, the fragment was melted by raising the temperature under the following conditions: denaturation at 95°C for 1 min, renaturation at 40°C for 1 min, and then melting that consisted of a continuous fluorescent reading from 60°C to 90°C at 25 acquisitions per °C.

The LunaProbes analysis was performed using the standard software included with the LightScanner96 instrument and analysis software (Idaho Technology, Inc., Salt Lake City, UT). Normalized melting curves showed the fluorescence signal against the temperature, and derivative plots showed the melting temperature peaks. The plasmids with known mutations were regarded as standard references. When the plots of samples were classified into the standard reference, they were identified as the same mutation of the standard.
